# Advances in the Treatment of Malaria

**DOI:** 10.4084/MJHID.2012.064

**Published:** 2012-10-03

**Authors:** Francesco Castelli, Lina Rachele Tomasoni, Alberto Matteelli

**Affiliations:** 1 Chair of Infectious Diseases, University of Brescia, Italy.; 2 University Division of Infectious and Tropical Diseases, University of Brescia and Spedali Civili General Hospital, Brescia (Italy).

## Abstract

Malaria still claims a heavy toll of deaths and disabilities even at the beginning of the third millennium. The inappropriate sequential use of drug monotherapy in the past has facilitated the spread of drug-resistant *P. falciparum*, and to a lesser extend *P. vivax*, strains in most of the malaria endemic areas, rendering most anti-malarial ineffective. In the last decade, a new combination strategy based on artemisinin derivatives (ACT) has become the standard of treatment for most *P. falciparum* malaria infections. This strategy could prevent the selection of resistant strains by rapidly decreasing the parasitic burden (by the artemisinin derivative, mostly artesunate) and exposing the residual parasite to effective concentrations of the partner drug. The widespread use of this strategy is somehow constrained by cost and by the inappropriate use of artemisinin, with possible impact on resistance, as already sporadically observed in South East Asia. Parenteral artesunate has now become the standard of care for severe malaria, even if quinine still retains its value in case artesunate is not immediately available. The appropriateness of pre-referral use of suppository artesunate is under close monitoring, while waiting for an effective anti-malarial vaccine to be made available.

## Introduction and Historical Outline

The correct management of clinical malaria cases is a complex issue that has to take into account different targets that may be differently prioritized according to the various clinical and epidemiological situations:

- to prevent progression of uncomplicated malaria patients to severe life threatening complications (*P. falciparum* but also *P. vivax*);
- to prevent mortality of patients with severe malaria (*P. falciparum* but also *P. vivax* and *P. knowlesi*);
- to prevent relapses when appropriate (*P. vivax, P. ovale*);
- to limit the spreading of the infection/disease in the population;
- to limit as much as possible the emergence of plasmodium resistant strains.

Considering the complex biological cycle of malaria plasmodia, the ideal drug to meet the *clinical* targets should have the following properties:

- to act rapidly against the replicating blood erythrocytic asexual forms, primarily schizonts, that are responsible for the clinical manifestation of the disease (parasitological cure);
- to act against liver hypnozoites, when appropriate (radical cure).

In endemic areas, furthermore, the ideal drug to meet the *epidemiological* targets should have the following properties:

- to act against the sexual forms (gametocytes) that are responsible for the transmission of the infection in the population via the vector mosquitoes; this gametocidal effect is time-sensitive because the appearance of sexual forms is delayed of several days from the clinical malaria attack;
- to avoid selecting plasmodia resistant strains (high resistance barrier).

After quinine selective therapeutical value against malaria plasmodia was first suggested by Francesco Torti in Italy (1712), no major advance in malaria chemotherapy occurred until the first decades of the XX century. At that time, and until recently, treatment of malaria attacks was based on the use of single drug regimens that were subsequently made available according to the emergence of resistance to the previously used molecules. *Pamaquine* and *chloroquine* were discovered in Germany in 1924 and 1934 respectively, followed by *proguanil* (England, 1944), *pyrimethamine* (England, 1952), *primaquine* (USA, 1956), *sulphonamides* (1960–66), *mefloquine* (USA, 1971–75) and *halofantrine* (1989).^1^

The value of monotherapy had been questioned since the early ’60, when *P. falciparum* with decreased sensitivity to chloroquine appeared in South-East Asia and Colombia and then quickly spread to virtually all *P. falciparum* endemic areas. Thereafter, the same occurred to all antimalarial drugs acting against *P. falciparum* and, to a lesser extent, against *P. vivax*.

At the end of the 20^th^ century, the strong anti-parasite efficacy of the long-known Chinese malarial remedy artemisinine and its derivatives was scientifically demonstrated, both on blood asexual and sexual forms (gametocytes). Furthermore, in line with other major infectious diseases such as tuberculosis and HIV infection, the value of combination treatment to lessen the chance of a natural resistant strain to emerge was clearly established and new combination treatments tested (atovaquone-proguanil, chlorproguanil-dapsone).

Artemisinin based combination treatments (ACT) underwent extensive randomized clinical trials that proved their superiority in fever and parasitological clearance times and clinical outcomes.^2^

At present, therefore, artemisinin-based treatment complies with most of the properties of the “ideal antimalarial drug” listed above and represents the standard of care of both complicated and uncomplicated malaria.^3^

## Drug resistance of malaria plasmodia

According to WHO, resistance to malaria drug is defined as “*the ability of a parasite strain to survive and/or multiply despite the proper administration and absorption of an antimalarial drug in the dose normally recommended*”.^3^

This is particularly worrying for *P. falciparum*, both because of its higher propensity to develop resistance and because of its intrinsic higher virulence and morbidity and mortality burden. Resistance (or lower sensitivity) to antimalarial drugs has also been observed in *P. vivax*, while it is extremely rare (if present at all) in the other species of Plasmodia.

The spread of resistance is classically a two-step process. First, a mutant clone spontaneously emerges in the replicating parasite population. This clone is usually less fit that the wild-type sensitive ones and tends to disappear, unless it is confronted with selective drug pressure able to kill sensitive parasites but not blood circulating resistant asexual forms that subsequently evolve to gametocytes with possible spread in the population (second step). This phenomenon is usually more likely to happen first in low transmission settings where most parasite-carrying patients are symptomatic and therefore subject to treatment.^3^ This is probably the reason why chloroquine and pyrimetamine resistance strains first appeared in South-East Asia in the early sixties before spreading to the African continent,^4^ also favoured by the suggested higher predisposition to mutation of Asian *P. falciparum* strains.^3^

The probability of a genetic resistance mutation to occur is a function of many factors, including (but not limited to) (i) the number of replicating parasites and (ii) the drug concentration they are exposed to. It is then easy to understand why the therapeutic use of single drugs with long half-life (such as chloroquine or even mefloquine) and long decreasing concentration tails has facilitated the selection of resistant plasmodia strains.

Similarly to other infectious diseases where resistance is a major challenge (tuberculosis, HIV infection, etc.), a combination strategy to limit resistance has been proposed in the ‘90s using a rapidly acting and potent drug able to achieve a fast reduction of parasitic burden (limiting the intrinsic probability of genetic mutation), with few residual parasites exposed to high concentration of the long-acting partner drug (thus limiting the selective potential of low drug concentration).^5^ The rapid acting component of these combinations, thereafter called Artemisinin Combination Therapies (ACT), have been identified to be artemisinin and its derivatives, that are now considered the cornerstone of malaria treatment.

Unfortunately, as a possible consequence of drug misuse both in combination and in monotherapy, resistance to artemisinin derivatives has been reported, once again in South East Asia,^6^ forcing the implementation of a Global Plan for Artemisinin Resistance Containment (GPARC) by the World Health Organization.^7^

## Artemisinin and artemisinin-based combination therapy (ACT)

Artemisinin is a sesquiterpene trioxane lactone extracted from the plant *Artemisia annua* obtained by the Chinese program named ‘Project 523’ in the 1970’s.^8^ Its derivates (artesunate, artemether, and arteether) act with a mechanism, still largely unknown^9^ that makes them almost a perfect *P. falciparum* killer. It’s effective on a broader age range of the parasite than do other antimalarial drugs (with considerable effect on ring stages and early immature gametocyte stages, but not on extra-erythrocytic forms - sporozoites, liver schizontes or merozoites).^10^ Artemisinin and its derivatives act very fast *via* the common active metabolite dihydroartemisinin with a very high killing rate: the parasite reduction ratio (PRR), representing the fractional reduction per each asexual life cycle (48 hours long), is in the order of 10^4^. Such activity profile would predict a radical cure (eradication of all parasite from the body) in 7–8 days even when baseline total parasite burden is >10^12^ (100,000/μl or 2% parasitemia).^11^ Non clinical observations^12^ show a good and fast absorption regardless the administration route mode (T_max_ 0.5–1 hour after oral assumption, while intramuscular injection leads to slower absorption and longer sustained plasma levels after repeated administrations with possible increased toxicity). Tissue distribution is good, with high brain penetration and selective carrier-mediated entry into infected erythrocytes where drug concentration is 100-fold greater than in uninfected erythrocytes. Artemisinin and its derivates are bio-transformed by cytocrome P450 into the active metabolite dihydroartemisinin, with the exception of artesunate, which is an ester of the latter and is converted by esterases. However, Achilles’ heel of artemisinin and its derivates is their very short half-life, ranging from 2 to 5 hours, while artesunate’s and artemether’s half-life are <1 hour and 2–4 hours respectively.^13^ Artemisinin has a time-dependent pharmacokinetic profile with decreased plasma drug level after five consecutive days of oral administration.^14^ Consequently, when artemisin derivatives are used alone, they require long (> 5 days) course of treatment to be fully effective, raising the problem of poor compliance in normal clinical setting. Artemisinin monotherapy is therefore burdened by high failure rate with recrudescence risk ranging between 25 and 50%.^15^,^16^

To overcome this problem, artemisinin drugs are now used in combination with other antimalarial drugs with longer half-lives: artemisinin combination therapy (ACT). The ACTs take advantage of the strong and fast initial activity of the artemisinin derivative and of the favorable pharmacokinetic properties of the companion drug that, after a short course treatment, continues to act on low level parasitemia until radical cure.^11^,^17^ With a 3-days course of artemisinin, as now recommended by WHO,^3^ a 90% reduction of parasite burden is obtained. Ideally the partner drug should be selected among still well-fitting anti-malarial drugs (ensuring at least a 80% cure rate by itself) and with a half-life not as long to expose replicating parasites to sub-therapeutical drug level that may favour the emergence of resistant parasites. For this reason, the choice of the companion drug might be different in Sub-Saharan Africa and in East Asia.

Currently, the ACTs are the most potent weapon in treating *falciparum* malaria and, from the public health perspective, to limit the spread of drug-resistant strains.^18^

In fact, as for treatment of tuberculosis, leprosy, HIV and many cancers, combining drugs with different mode of action and resistance mechanism, reduces the probability of selecting resistance to both drugs: it has been calculated that it could naturally happen in 1 over 10^24^ parasites, so once over 10,000 years, being 10^20^ the cumulative total parasite burden in humans each year.^5^ To delay the emergence of *P. falciparum* resistance to artemisinin derivatives, monotherapy is to be absolutely avoided both in paediatric and adult populations.^19^ In 2005, WHO issued a warning about the risk of emergence and spread of artemisinin resistance from the Greater Mekong sub-region, where evidence of a slower parasite clearance was emerging. A recent study from Cambodia^6^ has confirmed the spread of artemisinin resistance, previously reported in the western border of Thailand^20^ where artesunate–mefloquine combination has been the first-line treatment for *P. falciparum* malaria since 1994.

Low toxicity is generally attributed to artemisinins. Animal (on rats) studies have suggested toxicity on the haematopoietic system with reticulocytes reversible decrease, but clinical observations point out to a lower toxicity in malaria patients compared to healthy volunteers.^21^ Cardiotoxicity could be related to QTc prolongation that has been reported at significant level after high intramuscular doses of the oil-based artemether and artemotil in toxicological studies conducted in beagle dogs.^22^ However, in humans, QTc interval was unaffected by intravenous bolus therapeutic artesunate doses (2.4 mg/kg).^23^

Fatal neurotoxicity, associated with administration of artemether and arteether intramuscularly or artelinic acid orally, has been demonstrated in animals but only for long drug exposure that is not comparable to that obtained with therapeutical courses as recommended for humans.^24^

Recently, cases of late haemolysis after parenteral treatment with artesunate have been reported.^25^ This phenomenon, whose underlying mechanism is still largely unknown, had also been reported in vitro in the ‘80s^26^ and in the animal model,^27^ and it is more pronounced at high dose of artemisinin derivatives and requires longer follow-up of the patients.

## Uncomplicated *P. falciparum* malaria

At the end of the nineties, the World Health Organization (WHO) has promoted a series of clinical trials testing the efficacy of artemisinin-based combinations using various partner drugs (ACTs) in various continents to treat uncomplicated malaria patients.

A large bulk of clear evidence of the superiority of ACTs in achieving both parasitological (parasite clearance time) or clinical (fever clearance time, survival) end points^28^,^2^ has been accumulated in the following years.

Based on these convincing data, WHO now recommends the use of five common ACT combinations (table 1) as first treatment of uncomplicated *P. falciparum* malaria in endemic areas. With the exception of artesunate–sulfadoxine–pyrimethamine, the recommended combinations are now available as fixed-dose treatments, which are preferable because of improved ease of use and adherence. Since the few residual parasites surviving the potent and fast effect of the artemisinin component of the ACT are thereafter confronted to the action of the long-acting partner drug, their local sensitivity pattern to the latter is of paramount importance to select the appropriate ACT combination in different geographical settings. Nationally recommended guidelines should carefully consider local resistance patterns to select the ACT combination that has the best chances to remain active for as long as possible.

Even if relatively limited data exist regarding the pharmacokinetic properties of these drugs in pediatric population,^29^ ACTs are proposed as first line treatment in children too. To solve problems of swallowability, palatability and dosing, pediatric formulations have been recently developed (syrup, powder for suspension, dispersible tablets and granules) with some evidence of an efficacy comparable to conventional co-formulations (about 98% cure rate) and of a better gastrointestinal tolerability, leading to improved management.^30^ However, the evaluation of efficacy, safety and tolerability of administration of pediatric ACTs is still under study.^31^

Evidence of embryo-toxicity and lethality in animal studies^32^ justifies WHO prudence: for pregnant women ACTs are currently proposed as first-line treatment only in the second and third trimester; during early pregnancy, the use of an ACT is allowed only if the recommended treatments (a seven-day course of quinine plus clindamycin or quinine monotherapy if clindamycin is not available) is not available or has failed, being artesunate plus clindamycin the second-line treatment.^3^ Accidental exposures to artemisins in first trimester of pregnancy is being monitored^33^ but further studies are needed.

Provided that it has not be used for prophylaxis, *atovaquone-proguanil* (table 1) is also considered among the first-line options for travellers returning to non endemic area^34^,^3^ and for areas with confirmed artemisinin resistance. Atovaquone acts on the mitochondrial membrane potential,^35^ while proguanil interacts with parasite DNA synthesis, inhibiting plasmodial dihydrofolate reductase. Their synergistic action brings to a 98% cure rate^36^ and performs better than mefloquine.^37^ Although the limited data available suggest that the risk of birth defects associated with atovaquone-proguanil exposure do not exceed 3-times the one observed in the general population^38^, the drug is not currently recommended for use during pregnancy.

When ACT and atovaquone-proguanil are not available or contraindicated, a second line option could be oral *quinine plus clindamycin* or *doxycycline* (the latter not to be administered in pregnancy and in children below 8 years) but a 5–7 days treatment course is required with risk of poor adherence mainly linked to quinine-related cinchonism (deafness, ringing in the ear, nausea).

Even if no more recommended by WHO in monotherapy,^39^,^3^ *mefloquine*, a 4-quinoline methanol, is still considered an acceptable option to treat imported uncomplicated *P. falciparum* malaria in some western countries guidelines^40^,^41^ but not in others, mainly in relation to a higher rate of neurological adverse effects observed at treatment dosages.^42^,^43^

Treatment of *P. falciparum* uncomplicated malaria with *chloroquine* is accepted only in patients returning from those limited areas where *P. falciparum* remains sensible to this drug (Haiti, Dominican Republic, Middle East and Central America - north of Panama Canal), if ACTs are not available.

## Uncomplicated non *P. falciparum* malaria

It’s important to underline that, when *P.falciparum* can’t be excluded (co-infection cases; mixed species malaria), any case of uncomplicated malaria coming from areas where resistance is reported should be managed as a *P. falciparum* malaria, the more so considering that ACTs and atovaquone-proguanil are effective on blood stages of non-falciparum *Plasmodium* species.^18^,^44^–^47^

However, when *P. falciparum* infection is safely excluded, chloroquine remains the standard of care for *P. malariae*, *P. ovale* and for *P. vivax* malaria. A total dose of 25 mg/kg is recommended (10 mg/kg at T0, followed by 5 mg/kg after 6, 24 and 48 hours or, alternatively, 10mg/kg on first and second day and then 5 mg/kg on third day).^3^ However, *P. vivax* is showing decreasing sensitivity in some specific areas. Since first *P. vivax* chloroquine resistance report in 1989^48^ monitoring activity has shown resistant strains mainly in South-East Asia^49^ but also in East Africa^50^ and Central and South America^51^ even if the risk of treatment failure of this drug, as well as of primaquine, still remains mainly unknown. The experience with *P. falciparum* resistance allows a reasonable expectation of a deteriorating situation. Chloroquine is well tolerated and safe also in pregnant women and children.

As *P. ovale* and *P.vivax* imply a latent hepatic stage (hypnozoites), radical cure to avoid subsequent relapses requires an adjunctive course with a hypnozoites killing drug. Currently, the only molecules with significant activity against this parasite stage are the 8-amino quinolines (buloquine, primaquine, tafenoquine)^52^ whose mechanism of action is not well understood but is probably focused on damage of parasite mitochondrial membrane and interference with the parasite's DNA structure.^53^ Only primaquine is currently on the market since when it was first licensed by FDA in 1952 as an anti-malarial drug. The other 8-amino quinoline drugs are still under investigation and seem to possess better pharmacokinetic characteristics (tefenoquine has a longer half-life)^54^ and a safer profile (buloquine has a less oxidative toxicity)^55^ than primaquine. In fact, the main safety concern in primaquine use is the risk of severe intravascular haemolysis in patients with glucose-6-phosphate dehydrogenase deficiency (G6PDH), which can be life threatening for patients with Mediterranean B variant of the X-chromosome gene. Glucose-6-phosphate dehydrogenase activity is then to be mandatorily assessed before primaquine administration. The drug is contraindicated in cases of severe deficiency (WHO class I and II; ≤ 10% residual enzyme activity)^56^. In mild-to-moderate G6PDH deficiency (WHO class III; 10–60% residual activity) primaquine 0.75 mg base/kg body weight may be safely administered once weekly for 8 weeks;^3^ in patients without G6PDH deficiency (WHO class IV and V; > 60% enzyme activity) the conventional daily drug adult dosage is 0,25 mg/Kg body weight up to 15 mg/day for 14 days^57^ to be taken with food. The efficacy of such low primaquine doses (< 5 mg/kg total dose) in preventing *P. vivax* relapses is however geographically variable.^58^ The Centers for Disease Control and Prevention (CDC) and other Authors currently recommend to increase the adult dosage to 0.5 mg/kg of body weight daily (maximum 30 mg divided in 2 doses) for 14 days when treating Asian *P. vivax* strains.^41^,^59^,^60^ Primaquine is contraindicated in pregnant women irrespective of their G6PDH status because the fetus G6PDH status can’t be assessed with certainty and the risk of severe hemolysis and *hydrops fetalis* may not be ruled out. On the opposite, lactating women can receive the drug if both the mother’s and the child’s G6PDH activity is adequate. Although data are lacking, there is no evidence suggesting that children of any age with normal G6PDH activity do not tolerate the drug. However, some public health authorities suggest caution under various age limits, ranging between 1 month and 4 years.^59^

Furthermore, primaquine displays a synergistic effect against blood stages when combined with chloroquine. However, the use of an ACT regimen (with exclusion of artesunate plus sulfadoxine-pyrimethamine) seems to be more appropriate in those areas with *P. vivax* chloroquine resistance where G6PDH activity testing is not easily available.^3^,^61^ Also in this case, however, only a primaquine course guarantees a radical cure from hypnozoites.

*Plasmodium knowlesi* is the newcomer among human malaria agents. It is microscopically undistinguishable from *P. malariae* and may even be misdiagnosed as *P. vivax* or *P. falciparum* (early trophozoites).^62^ Uncomplicated *P. knowlesi* infection may be cured by chloroquine as other non *falciparum* malaria. However no official guideline to treat *P. knowlesi* infection is currently available and there is evidence that other drugs, including mefloquine, quinine, atovaquone/proguanil and sulphadoxine-pyrimethamine may be active against *P. knowlesi*.^63^

## Severe malaria

Severe malaria, as defined by clinical or laboratory criteria as shown in table 2 or by high parasitemia (≥ 2% in non immune patients; ≥ 5% in patients in endemic areas), is usually caused by *Plasmodium falciparum* infection. However, an increasing body of evidence indicates that other Plasmodium species, in particular *P. vivax*, may induce severe forms of the infection.^64^,^65^ Case-fatality ratio is high (around 10%)^66^ especially among children and even after adoption of intravenous recommended anti-malarial regimens. Patients can deteriorate very quickly with greatest risk of death in the first 24 hours, especially in case of pediatric patients,^67^ so that a pre-referral treatment is recommended when appropriate intravenous therapy is likely to be delayed for more than 6 hours.^68^

Severe malaria should be regarded as a medical emergency and possibly managed in intensive care units (ICU) in order to assure adequate monitoring and treatment of organic dysfunctions.^69^–^71^

The mainstay of severe malaria therapy, irrespective of the responsible *plasmodium* species, is a prompt, parenteral, effective anti-malarial treatment with the primary goal of preventing death and disabilities and, only secondarily, recrudescences. Since 2006 WHO recommends intravenous artesunate as first line regimen, preferred to intravenous quinine whenever possible. After multicenter trials have established significant superiority of artesunate over quinine both in South-East Asia^72^ and in children in Africa,^73^ systematic reviews^74^ have demonstrated its effectiveness in reducing case fatality rates regardless of age and geographic differences (RR 0.71). For this reason i.v. artesunate is now considered the standard of care even in the absence of an international drug regulatory authority registration and even against the risk of reduced availability outside Asia. Of notice, the non-GMP (Good Manufacturing Practices) i.v. artesunate produced by Guilin Pharmaceutical Company Ltd., Shanghai, China (the one used in the SEQUAMAT and AQUAMAT trials) has recently been prequalified by WHO.^75^ Considerable efforts are currently being made to make a GMP i.v. Artesunate formulation available for clinical use in western countries. In the U.S. the Walter Reed Army Institute of Research (WRAIR) has undergone Phase I trials^76^,^77^ of a formulation currently approved by FDA as an investigational drug, that may be directly requested to CDC.^78^ However, also pharmaceutical companies (e.g. Sigma-Tau, Italy) are investing in GMP-standard i.v. artesunate production programmes and on 2007 the European Medicines Agency has assigned the Orphan Medicinal Drug Designation to the drug.^79^

In Europe, only French, Dutch and Belgian National Health Agencies have temporarily authorized the use of i.v. artesunate within a named patient programme.^80^ Malacef ® is the Guilin i.v. artesunate, imported and distributed after quality control by a Dutch company (ACE Pharmaceuticals). Where there is no official authorization, however the use of non –GMP i.v. artesunate may still pose problems under the legal point of view. To overcome this obstacle, a treatment combining market authorized i.v quinine and WHO recommended i.v. prequalified artesunate has been used with satisfactory efficacy and safety profile.^81^,^82^

The absence of randomized controlled trials to support i.v. artesunate superiority in imported severe malaria cases in non-endemic areas still causes some perplexity.^83^ However, both in US by CDC and in Europe by TropNetEurope, a close efficacy and safety monitoring is carried out.^84^,^85^

If the SEAQUAMAT study has shown a better safety profile of artesunate when compared with quinine with statistically (p=0.009) significant reduction of hypoglycemia, systematic analysis of randomized trials^74^ has pointed out a higher non-statistically significant rate of neurological problems at discharge in patients, especially children, treated with artesunate. This fact may be related to the increased survival of cerebral malaria cases and anyway neurologic sequelae where not permanent. Both SEAQUAMAT and AQUAMAT studies, however, failed to capture another safety concern that is now emerging by observational studies reporting post-treatment haemolysis, mainly in imported severe malaria cases.^84^–^87^ Patients should be carefully monitored for at least one month after treatment because haemolytic anemia can appear longer after artesunate clearance (median elimination T ½ is 0.25 [0.11 – 1.82] hours).^88^ The precise mechanisms underlying such phenomenon are unclear at the moment, nor risk factors are known. As a precautional measure, it could then be prudent to limit the use of i.v. artesunate to the shorter possible necessary (however keeping a minimum of 24 i.v. infusion) in order to exploit its high parasite clearance activity during the first 24 hours and to avoid long unnecessary i.v. treatments. The intra-venous treatment should be followed by a full course of an effective oral anti-malarial treatment: WHO suggests effective ACT (artesunate plus amodiaquine or artemether plus lumefantrine or dihydroartemisinin plus piperaquine) or artesunate (plus clindamycin or doxycycline) or quinine (plus clindamycin or doxycycline) and does not recommend the use of mefloquine because of the increased risk of neuropsychiatric events after cerebral malaria.^3^

Where intravenous parenteral treatment with artesunate is not immediately available, i.v. quinine should be used (table 3). In remote settings, far from health care facilities that could ensure intravenous treatment, pre-referral intra muscular quinine, artemether or artesunate and, even easier to administrate, rectal artesunate are currently recommended by WHO for children.^68^ In particular, a placebo controlled trial has shown superiority (p=0.0013) of rectal artesunate over placebo to prevent death or permanent disability (RR 0.49).^89^ Even if the trial has been the subject of controversial debate as to its methodological approach,^90^ it has the great merit of remarking the urgency of immediate treatment in cases of severe malaria. Some concern may arise as to the risk that encouraging the use of rectal artesunate in monotherapy could impact on resistance pattern. Anyway evaluation of efficacy and appropriateness of this strategy is ongoing.^91^

## Figures and Tables

**Table 1:** Recommended regimens for the treatment of uncomplicated *P. falciparum* malaria (various sources) §

Table 1A: First line regimens						
Compound (not exhaustive)	Manufacturers (not exhaustive)	Formulations	Dosage (adult)	Dosage (child)	Notes	Pregnancy
Arthemether-lumefantrine (Riamet ® - Coartem ®)	Novartis/Chinese Academy of Medical Military Sciences/MMV	Tablet (adult) and Dispersible (child): 20mg/120 mg	4 tablets for 6 doses (0-8-24-36-48-60h)	5–14 kg: 1 for 6 doses 15–24kg: 2 for 6 doses 25–34kg: 3 for 6 doses (0-8-24-36-48-60h)	With food Reduced efficacy in Cambodia and border regions of Thailand	II and III trim (I trim only if no alternative regimens)
Dihydroartemisi nin–piperaquine (Eurartesim ®)	Sigma-Tau/MMV/Pfizer	Tablet (adult): 320/40 mg Crushed and Dispersible (child): 160/20 mg	36–74kg: 3 tablets 74–100kg: 4 tablets once daily for 3 days	5–7 kg: ½ tablets 7–13kg: 1 tablets 24–36kg: 2 tablets once daily for 3 days	Fasting	II and III trim (I trim only if no alternative regimens)
Artesunate–amodiaquine (Coarsucam ®)	Sanofi-Aventis/DNDi/MMV)	Dispersible tablet: 25/67.5 mg 50/135 mg 100/270 mg	>36 kg 200/540mg once daily for 3 days (4/10mg/kg)	4.5–9 kg: 25/67.5 mg 9–18 kg: 50/135 mg 18–36kg: 100/270 mg once daily for 3 days	Not with high fatty food	only if no alternative regimens
Artesunate–mefloquine (Artequin ®, Mefliam-plus®)	Mepha, ASMQ; Farmanguinhos/ DNDi/Cipla	Crushed tablet: 25/55(=50) mg 100/220 (=200) mg	200/440mg once daily for 3 days	5–8 kg: 25/55mg 9–17kg: 50/110mg 18–29kg: 100/220mg >29kg: 200/440mg once daily for 3 days	With food	II and III trim (I trim only if no alternative regimens)
Artesunate–sulfadoxine–pyrimethamine (Altinate ®, Larinate ®, Artescope ®)	Allenge, Intima, Guilin	Tablets, various co-formulations	Artesunate 4 mg/kg/day for 3 days Sulfadoxine/pyrimetamine 25/1.25 mg/kg on day 1			II and III trim
Atovaquone-proguanil (Malarone ®)	GlaxoSmithKline	Tablet (adult): 250/100 mg Tablet (child): 62.5/25 mg	4 tablets once daily for 3 days	5–8kg: 1 tablet 62.5/25 9–10Kg: 2 tablets “ “ 11–20Kg: 1 tablet 250/100 21–30kg: 2 tablets “ “ 31–40kg: 3 tablets “ “ >40 kg: 4 tablets “ “	With fatty meal. Use for travellers	Only if no alternative regimens available
Table 1B: First line regimens (various sources)						
Compound (not exhaustive)	Manufacturers (not exhaustive)	Formulations	Dosage (adult)	Dosage (child)	Notes	Pregnancy
Quinine^ + Clindamycin			10mg/kg thrice daily + 10mg/kg thrice daily for 7 days	10mg/kg thrice daily + 10mg/kg thrice daily for 7 days	Off-label use	OK
Quinine^ + Doxycycline			10mg/kg thrice daily + 200 mg daily for 7 days	Contraindicated if <8 years	Off-label use	Controindicated
Mefloquine (Lariam ®, Mephaquin ® or Mefliam ®)	Roche, Mepha, Cipla	Tablet: 250mg	45–60kg: 5 tablets (3+2) >60kg: 6 tablets (3+2+1) 6–8 hours apart	20–25mg/kg divided in 1–3 doses 6 hours apart: 5–10 kg: ½–1 tablet 10–20kg: 1–2 tablets 20–30kg: 2–3 tablets 30–45kg: 3–4 tablets	With food Not suitable for SE-Asia	II and III trim; I trim only if no alternative regimens

§ : please refer to drug package instruction before use

^ Dosages are for Quinine dihydrocloride. Equivalent doses for available quinine salts are as follow: quinine base 100 mg= quinine bisulfate 169 mg= quinine dihydrochloride 122 mg= quinine hydrochloride 111 mg=quinine sulfate 121 mg=quinine gluconate 160 mg.

**Table 2:** Conditions defining severe malaria case in plasmodium infection (ref: 3). Severe malaria is defined, in the absence of other obvious cause, when *P. falciparum* asexual parasitaemia is accompanied by one or more of the following clinical or laboratory features.

*Clinical features:*
impaired consciousness or unrousable coma prostration, i.e. generalized weakness so that the patient is unable to walk or sit up without assistance failure to feed multiple convulsions – more than two episodes in 24 h deep breathing, respiratory distress (acidotic breathing) circulatory collapse or shock, systolic blood pressure < 70 mm Hg in adults and < 50 mm Hg in children clinical jaundice plus evidence of other vital organ dysfunction haemoglobinuria abnormal spontaneous bleeding pulmonary oedema (radiological)
*Laboratory findings:*
hypoglycaemia (blood glucose < 2.2 mmol/l or < 40 mg/dl) metabolic acidosis (plasma bicarbonate < 15 mmol/l) severe normocytic anaemia (Hb < 5 g/dl, packed cell volume < 15%) haemoglobinuria hyperparasitaemia (> 2% or 100 000/μl in low transmission areas or > 5% or 250 000/μl in high stable malaria transmission areas) hyperlactataemia (lactate > 5 mmol/l) renal impairment (serum creatinine > 265 μmol/l).

**Table 3:** Treatment of severe malaria §

	Dosage / body weight		Notes

Parenteral Drug			

i.v. Artesunate	2.4 mg/kg at 0,12,24 then once a day		Available as single-dose vial containing drug as a sterile dry-filled powder and a single-dose vial of a buffer solution to be reconstituted in a clear colorless 10 mg per mL solution. Adding 5 ml of normal saline solution it can be administered direct i.v. over 1 to 2 minutes into an established i.v. line.

i.v. Quinine	20 mg*/kg (loading dose) then 10 mg/kg at 8-h intervals * Quinine doses are usually prescribed as quinine dihydrochloride salt (10 mg of salt = 8.3 mg of quinine base).		contra-indicated if previous blackwater fever or quinine hypersensitivity or cardiac arrhythmia are known avoid loading dose if oral quinine or mefloquine has been given within 24 hours or if QT interval at baseline ECG is >25% each dose in 10 ml/kg of saline or 5% dextrose solutions (maximum concentration 60mg/ml) rate-controlled infusion not exceeding 5 mg salt/kg body weight per hour (2–4 hrs) never by intravenous bolus injection → lethal hypotension reduce quinine dose to 5–7 mg/kg if infusion last for more than 48 hrs or if pt develops renal failure monitor blood glucose levels and electrocardiographic features

Pre-referral drugs			

Suppository ** artesunate ** 50,100,400 mg	5 to 8 kg	1supp. 50 mg	given once and followed as soon as possible by parenteral therapy
	9 to 19 kg	1supp.100 mg	
	20 to 29 kg	2supp 100 mg	
	30 to 39 kg	3supp 100 mg	
	40 to 59 kg	1 supp 400 mg	
	60 to 80 kg	2 supp 400 mg	
	>80 kg	3 supp 400 mg	

i.m. Quinine	10 mg/kg		Dilute 300 mg quinine (usually corresponding to 1 ml) into 5ml of sterile water for injection or saline (not dextrose) in the same syringe, that now contains 50mg quinine/ml

i.m. Artemether	3.2mg/kg		

§ : please refer to drug package instruction before use

Parenteral antimalarials should be administered for a minimum of 24 h, once started. When parasitemia has decreased to less than 1% and patient can tolerate oral medication, treatment should be completed by giving a complete course of: (i) artemether plus lumefantrine, (ii) artesunate plus amodiaquine, (iii) dihydroartemisinin plus piperaquine, (iv) artesunate plus sulfadoxine-pyrimethamine, (v) artesunate plus clindamycin or doxycycline, (vi) quinine plus clindamycin or doxycycline
